# First Complete Coding Sequence of a Venezuelan Equine Encephalitis Virus Strain Isolated from an Equine Encephalitis Case in Costa Rica

**DOI:** 10.1128/MRA.00672-19

**Published:** 2019-09-05

**Authors:** Bernal León, Carlos Jiménez, Rocío González, Lisbeth Ramirez-Carvajal

**Affiliations:** a Biosecurity Laboratory (LSE), Animal Health National Service (SENASA), Heredia, Costa Rica; b Virology Laboratory, Veterinary Medicine School, Tropical Disease Research Program, Universidad Nacional (UNA), Heredia, Costa Rica; c Pathology Laboratory, LSE, Animal Health National Service (SENASA), Heredia, Costa Rica; d Department of Biomedical Sciences, Section of Anatomic Pathology, Cornell University, Ithaca, New York, USA; e Diagnostic Department, Animal Health National Service (SENASA), Heredia, Costa Rica; Portland State University

## Abstract

The first complete coding sequence of the Venezuelan equine encephalitis virus IE, isolated from a Costa Rican mare with severe encephalitis, was confirmed by histological and viral whole-genome analyses. The isolated virus grouped in the Pacific cluster.

## ANNOUNCEMENT

**FIG 1:**
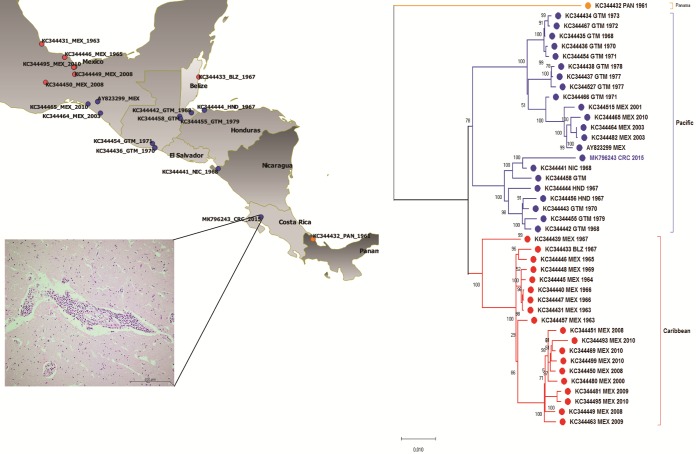
Phylogenetic relationship of IE-VEEV. Color coding in the phylogenetic tree refers to classification of the clades, with orange for Panama, red for the Caribbean group, and blue for the Pacific group. A map made with QGIS 3.6 (17) depicts the same color scheme for the geographical distribution of the sequences (from southern Mexico to northern Panama). In the case of the sequence KC344441, the location of the sample is an estimate based on the available information in a public database. In the case of the remaining sequences, the location refers to the town or state cited in the database. Sample LSE9010-15 shows the collection site. (Inset) Histological hematoxylin and eosin (H&E) staining section from the basal ganglia of the VEEV-infected mare with encephalitis.

Venezuelan equine encephalitis virus (VEEV) is an emerging infectious zoonotic agent in Latin America which is transmitted by mosquitoes (1). The VEEV genome is a single positive 5′-capped, 3′-polyadenylated RNA strand of ∼11.5 kb, which encodes four nonstructural proteins (NSP1 to -4) and the structural proteins processed from a precursor polyprotein organized as NH2-capsid protein-E3-E2-6K-E1-COOH (2). VEEV is 1 of the 31 species of the Alphavirus genus within the *Togaviridae* family (3, 4). Importantly, the VEEV subtype I was divided into four variants. Variants IAB and IC are considered virulent epizootic variants (5), and ID and IE are nonpathogenic enzootic variants (6). The members of VEEV subtype I are found in Mexico (5), Central America (7), and South America (1, 8, 9). Subtype IE was considered an avirulent strain in Mexico and Central America until 1993, when two outbreaks in Chiapas and Oaxaca in the southern Pacific coast of Mexico caused the deaths of 63 and 12 horses, respectively. Partial nucleotide sequence analysis revealed few nucleotide differences between epizootic and enzootic IE strains in those outbreaks (5). Here, we report the first complete *Alphavirus* coding sequence in Costa Rica and the first VEEV IE strain isolated from a clinical case. A horse brain sample, identified as LSE9010-15, was analyzed at the Animal Health National Service Laboratory (SENASA-LANASEVE) and showed severe lymphoplasmatic and neutrophilic encephalitis, characterized by moderate to severe multifocal perivascular infiltration of lymphocytes, plasma cells, and scant neutrophils (Fig. 1, inset). The sample tested negative for rabies and positive for VEEV by an in-house reverse transcription-PCR (RT-PCR). Briefly, an occipital cortex sample was disrupted and homogenized using a TissueLyser II (Qiagen, USA) and clarified by centrifugation, and the supernatant was extracted with the QIAamp cador pathogen minikit (Qiagen). Total nucleic acids were used for a one-step retrotranscription PCR kit (Qiagen) with the primer pair FW, 5′-CCCAAAATGGAGAAAGTTCAC-3′, and RV, 5′-GCCCAGTTGGTAGAGTATGATG-3′, at 0.54 μM. An RNase inhibitor (0.04 U/μl) was added to a 12.5-μl final reaction mixture. The cycle program used was 30 min at 56°C; 15 min at 95°C; 35 cycles of 20 s at 95°C, 20 s at 57°C, and 40 s at 72°C; and 5 min at 72°C. An amplicon of 605 bp was visualized in a 1% agarose gel stained with GelRed (Biotium, USA).

The supernatant of a single passage on Vero E6 cells infected with VEEV was clarified by centrifugation and extracted with TRIzol LS (Invitrogen, USA) (10). RNA quality and quantity were determined using a NanoDrop spectrophotometer (Thermo Fisher, USA) and a Quantus fluorometer (Promega, USA). cDNA synthesis was conducted using random hexamers and the SuperScript III kit per the manufacturer’s instructions (Invitrogen). cDNA was treated with Klenow DNA polymerase (Applied Biosystems, USA) and RNase H (Invitrogen) at 37°C for 1 h 10 min at 75°C. Double-stranded DNA (dsDNA) was purified using AMPure XP magnetic beads (Beckman Coulter, Indianapolis, IN, USA). Genomic libraries were prepared using a commercial kit (Nextera XT DNA library; Illumina, USA) following the manufacturer’s instructions. Quality, quantity, and fragment size distribution of nucleic acids were evaluated with a NanoDrop spectrophotometer, a Quantus fluorometer (Promega), and a QIAxcel system (Qiagen, USA), respectively. The library was normalized, denatured, diluted to 2 nM, and sequenced on an Illumina MiSeq platform using a paired-end (2 × 250-bp) protocol. Run quality was assessed using the Sequence Analysis Viewer (Illumina). Read pre- and posttrimming quality was assessed using FastQC version 0.11.5 (11). Quality trimming was conducted on CLC Genomics Workbench version 10.1.1 (CLC bio, Qiagen, Denmark). The total number of sequence reads was 426,298, and after quality trimming, the number of reads was 421,551. *De novo* assembly and scaffolding were conducted using SPAdes version 3.10.1 (12) in careful mode and with default parameters. The longest scaffold was subjected to a BLAST search against the NCBI viruses database (taxid 10239) (13). The longest scaffold was aligned to VEEV's closest matches in the virus database (GenBank accession numbers KC344441 and KC344515) using Clustal Omega (14). The alignment was visually inspected and revised to generate the draft genome (size, 11,440 bp; 49.2% GC content) and transfer annotation. Quality-trimmed reads were mapped using the default parameters of CLC Genomics Workbench version 10.1.1 (CLC bio) to the draft genome (mapped read count, 328,101 reads; minimum coverage, 1×; maximum coverage, 7,795×; average coverage, 3,843×). LSE9010-15 shared ∼98% nucleotide identity with the Nicaragua isolate (KC344441) (13). Last, the nucleotide sequence of the LSE9010-15 genome was aligned to 41 IE-VEEV sequences downloaded from GenBank/DDBJ/ENA version 10.8 (15) using Clustal Omega (14). The phylogenetic tree was created with MEGAX version 10.0.2 (16), with a bootstrap of 1,000, using the maximum likelihood method and GTR+G substitution model. The phylogenetic tree confirmed three separate clusters proposed previously (5). The genomic sequence of sample LSE9010-15 (MK796243) was related to the Pacific clade (Fig. 1).

The sequences used in this study included those from Panama (KC344432), the Caribbean (KC344449, KC344457, KC344440, KC344447, KC344469, KC344450, KC344445, KC344433, KC344439, KC344431, KC344448, KC344446, KC344463, KC344495, KC344481, KC344493, KC344480, KC344451, and KC344499), and the Pacific (AY823299, KC344434, KC344435, KC344436, KC344437, KC344438, KC344441, KC344442, KC344443, KC344444, KC344454, KC344455, KC344456, KC344464, KC344465, KC344466, KC344467, KC344482, KC344515, KC344527, and KC344458).

### Data availability.

The genome sequence of Venezuelan equine encephalitis virus has been deposited in NCBI GenBank under the accession number MK796243 and BioProject number PRJNA546500.
